# Antiaflatoxigenic activity of *Carum copticum* essential oil

**DOI:** 10.1007/s10311-013-0439-x

**Published:** 2013-10-06

**Authors:** Mohammad Reza Rezaei Kahkha, Saeed Amanloo, Massoud Kaykhaii

**Affiliations:** 1 School of Health, Zabol University of Medical Sciences, 98616-15881 Zabol, Iran; 2 Department of Chemistry, Faculty of Sciences, University of Sistan and Baluchestan, Zahedan, Iran

**Keywords:** Aflatoxin, Essential oil, *Carum copticum*

## Abstract

Plants are unique sources of useful metabolites. Plant essential oils display a wide range of antimicrobial effects against various pathogens. Here, we studied the essential oil from the seeds of *Carum copticum*. We monitored aflatoxin by high-performance liquid chromatography. Results show that *Carum copticum* essential oil inhibits *Asergillus parasiticus* growth and prevents aflatoxin production. The half-maximal inhibitory concentration (IC_50_) is 127.5 μg mL^−1^ for aflatoxin B_1_ and 23.22 μg mL^−1^ for aflatoxin G_1_. Our findings show that *Carum copticum* essential oil is a potential candidate for the protection of foodstuff and feeds from toxigenic fungus growth and their subsequent aflatoxin contamination.

## Introduction

Aflatoxins are closely related toxic secondary metabolites produced by *Aspergillus flavus*, *Aspergillus parasiticus, Aspergillus nomius* and *Aspergillus tamarii* (Fooladi and Farahnaky 2003). There are four principal types of aflatoxins, namely B1, B2, G1 and G2. They are extremely potent, naturally occurring hepatocarcinogens (Henry et al. 1999). Considerable attention is being focused on aflatoxin control in agricultural produce because of health risks involved in the consumption of contaminated agricultural commodities. Prevention of mold growth on agricultural produce offers the best means of aflatoxin control because decontamination of foods and feeds containing preformed toxin is a challenging proposition. According to Rustom (1997), occurrence of mold growth is often unavoidable and still remains a serious problem in food commodities, emphasizing the need for a suitable detoxification protocol. Numerous attempts have been made to detoxify aflatoxin in feedstuff while ensuring the safety and nutritional adequacy of the produce (Keyl and Booth 1971). However, no single method of detoxification has completely solved the problem. Hence, an efficient method to remove aflatoxin either completely or to acceptable low levels while retaining the nutritional value of food commodity will be an attractive alternative.

In recent years, the essential oils and extracts of several plant species have become popular as fungi toxicants. Plants are considered as unique sources of useful metabolites. The plant essential oils received major considerations with regard to possessing a wide range of antimicrobial effects against different groups of pathogenic organisms (Bakkali et al. 2008). So, essential oils with antimicrobial activity are potential candidates, as natural antimicrobial preservatives, that can be used in controlling microbial food contaminations. Different plant preparations including the essential oils and extracts prepared from leaves, roots and seeds have successfully been used as potential inhibitors of growth and/or aflatoxin production by *A. flavus* and *A. parasiticus* (Mahmoud 1994; Mahoney et al. 2000; Rasooli and Razzaghi-Abyaneh 2004; Razzaghi-Abyaneh et al. 2005). These plants may be useful for controlling aflatoxin contamination of crops in field conditions.

*Carum copticum* seeds are used as a common household remedy for cholera, colic, diarrhea, dyspepsia, hypertension, asthma and hepato-biliary complications (Avesina 1985). The plant is known to possess antiallergic, antibacterial, anthelmintic, antifungal (Dubey and Mishra 1990), hypocholesterolemic, bronchodilator (Boskabady et al. 1998) and cholinergic activities. The phytochemical studies on Carum seeds have revealed the presence of multiple constituents including steroptin, ecumene, thymene, amino acids such as lysine and threonine, calcium, iron, starch, tannins and dietary fiber. The seeds also contain essential oil (2–3 %), which contain thymol (40–50 %), *γ*-terpinene, *p*-cymene, *α*-pinene, *β*-pinene and carvacrol (Ballba et al. 1973). Thymol is a strong germicide, antispasmodic agent and a known antifungal agent (Mahmoud 1994). Antimicrobial (Kavoosi et al. 2013) and antifungal activity of essential oil and alcoholic extract of *Carum copticum* (Ghahfarkhi et al. 2008) was known already. Also, it showed activities against food-borne and nosocomial pathogens (Zomorodian et al. 2011). So, it was interesting to study its activity against fungal growth *A. parasiticus* NRRL 2999. In this study, EO of *Carum copticum* was evaluated with regard to its ability to inhibit growth and aflatoxins (AFB1 and AFG1) production by *A. parasiticus* species.

## Experimental

### Materials and methods

The *Carum copticum* seeds were purchased from a local market. All chemicals used were of analytical grade and were obtained from Merck Chemicals Co., Germany. Aflatoxigenic Aspergillus parasiticus NRRL 2999 was used as a source of aflatoxins. Aflatoxigenic activity of fungal strain of Aspergillus parasiticus was known for many years (Meier and Marth 1977). It produces all 4 major aflatoxins, i.e., B1, B2, G1 and G2. The strain was maintained on potato dextrose broth slants by regular subculturing.

### Preparation of *Carum copticum* extract

Twenty-five grams of *Carum copticum* was steam distilled for 90 min at a Clevenger-type apparatus. The extraction was carried out for 120 min in 500 mL of water. The extracted essential oils were kept in dark at 4 °C before use.

### Microbioassay

Aflatoxigenic *A. parasiticus* NRRL 2999 was cultured on potato dextrose broth in six-well flat-bottomed microplates in the presence of *c. copticum* EOs. The culture medium was added to the microplates in amount of 5 mL well^−1^ and then inoculated with fungal spore suspension (5 × 10^6^ spore well^−1^) prepared in distilled water. Different concentrations of *C. copticum* essential oils (1, 10, 50, 100, 500 and 1,000 μg mL^−1^) prepared in methanol were added to the test wells. The control wells were treated in the same manner with the test wells except that they did not contained *C. copticum* essential oils. The microplates were incubated for 7 days on a rotary shaker at temperature of 28 ^o^C.

All contents of each well including the culture media and fungal biomass were filtered through a thin layer of cheesecloth and thoroughly washed with distilled water. A known weight of mycelia was placed in a container and allowed to dry at 80 ^o^C till a constant weight was obtained.

### Aflatoxin assay

The toxin was analyzed qualitatively for the presence of aflatoxins by thin-layer chromatography (TLC) using silica gel 60 F_254_ plates under ultraviolet light (maximum intensity at wavelength of 365 nm). The amount of toxin detected by TLC was 10 ng g^−1^.

### Quantitative analysis

Aflatoxin content of cultures was measured using an HPLC (Cecil UV–VIS system, England) equipped with a UV–Vis detector according to Razzaghi-Abyaneh et al. (2007). Fifty microliters of sample (culture filtrate) was injected into the HPLC column (ACE 5-C_18_; 4.6 × 150 mm) and eluted at a flow rate of 1 mL min^−1^ by water/acetonitrile/methanol (60:25:15, v/v/v) solvent mixture. The amount of aflatoxin B1 (AFB1) and aflatoxin G1 (AFG1) was detected at a wavelength of 365 nm by comparison of under-curved area of unknown samples with authentic standards treated in the same manner. The retention times of AFB1 and AFG1 were 5.0 and 6.3 min, respectively.

## Results and discussion

### Effect of plant essential oils on *A. parasiticus* growth

**Fig. 1:**
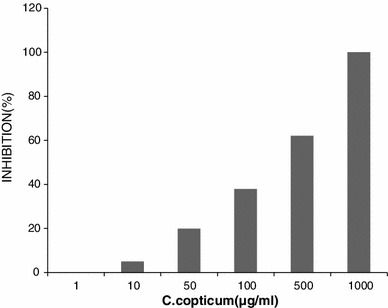
Effect of *C. copticums* essential oils on *A. parasiticus* NRRL 2999 growth in microbioassay (All data points are the average of 3 experiments performed in triplicate.)

As illustrated in Fig. 1, the inhibitory *C. copticums* essential oils show strong capabilities to suppress *A. parasiticus* grown in microplates. Generally, the inhibitory effect of essential oils increased in proportion to their concentrations and reached to a maximum in the final concentration of 1,000 μg mL^−1^. In this concentration, *C. copticum* showed the highest growth inhibition (100 %).

### Effect of plant EOs on aflatoxin production

**Fig. 2:**
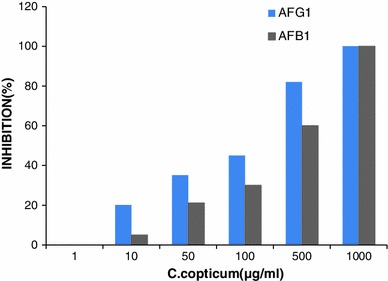
Inhibitory effects of *C. copticums* essential oils on production of aflatoxins B1 (*gray* *columns*) and G1 (*blue* *columns*) by *A. parasiticus* NRRL 2999 (All data points are the average of 3 experiments performed in triplicate.)

As indicated in Fig. 2, *C. copticums* essential oils also were able to suppress aflatoxin production by *A. parasiticus*. In the final concentration of 1,000 μg mL^−1^, *C. copticum* showed the highest growth inhibition (100 %). Minimum inhibitory concentration (MIC) was found to be 0.87 μg mL^−1^. As indicated in Table 1, the IC50 values of *C. copticums* for aflatoxin B1 were calculated as 127.5 μg mL^−1^, where as for aflatoxin G1, it is 23.2 μg mL^−1^ (Table 1).

**Table 1:** Inhibition percentage and IC_50_ values of essential oils of *C. copticum* for aflatoxins produced by *A. parasiticus* NRRL 2999

EOs concentration (μg mL^−1^)	Aflatoxin B1		Aflatoxin G1	
	IC_50_ **(**μg mL^−1^)	Inhibition (‰)	IC_50_ (μg mL^−1^)	Inhibition (‰)
10	90	127.5	85	23.22
50	80		75	
100	75		60	
500	50		15	
1,000	ND		ND	

These results clearly show that the essential oil of *Carum copticum* may have the potential to be used as natural preservatives in controlling aflatoxin contamination of foods, feeds and agricultural commodities in practice. A comprehensive ecological study is needed for evaluating the effectiveness of these inhibitory plants and their effective constituents in field condition. In addition, antimicrobial and antioxidant activities of *Carum copticum* should be considered by other scientists, because the combination of these properties may act in a synergistic manner by reducing lipid oxidation and *Carum copticum* could be a good candidate for extending the shelf-life of perishable foods.

## Conclusion

A novel method of toxin inactivation using aqueous *Carum copticum* extract has been described in the present work. The essential oil from *C. copticum* has effectively suppressed fungal growth. *C. copticum* was used as inhibitor of aflatoxin production by *A. parasiticus*. This plant suppressed aflatoxin B_1_ and aflatoxin G_1_ production dose dependently, consistent with its inhibitory effects on fungal growth. From data on antifungal activity of thymol and carvacrol as effective constituents of *Satureja hortensis* essential oil (Razzaghi-Abyaneh et al. 2008), it can be concluded that thymol, the main essential oil constituent of *C. copticum*, is responsible for inhibiting growth and aflatoxin production by the fungus. Our results clearly show that the essential oil of *C. copticum* may have the potential to be used as natural preservatives in controlling aflatoxin contamination of foods, feeds and agricultural commodities in practice.
